# Requirements for Driving Antipathogen Effector Genes into Populations of Disease Vectors by Homing

**DOI:** 10.1534/genetics.116.197632

**Published:** 2017-02-02

**Authors:** Andrea Beaghton, Andrew Hammond, Tony Nolan, Andrea Crisanti, H. Charles J. Godfray, Austin Burt

**Affiliations:** * Life Sciences, Imperial College, Silwood Park, Ascot, Berkshire SL5 7PY, United Kingdom; † Life Sciences, Imperial College, South Kensington, London SW7 2AZ, United Kingdom; ‡ Department of Zoology, University of Oxford, Oxford OX1 3PS, United Kingdom

**Keywords:** gene drive, population genetic engineering, pest control, homing, malaria

## Abstract

There is a need for new interventions against the ongoing burden of vector-borne diseases such as malaria and dengue. One suggestion has been to develop genes encoding effector molecules that block parasite development within the vector, and then use the nuclease-based homing reaction as a form of gene drive to spread those genes through target populations. If the effector gene reduces the fitness of the mosquito and does not contribute to the drive, then loss-of-function mutations in the effector will eventually replace functional copies, but protection may nonetheless persist sufficiently long to provide a public health benefit. Here, we present a quantitative model allowing one to predict the duration of protection as a function of the probabilities of different molecular processes during the homing reaction, various fitness effects, and the efficacy of the effector in blocking transmission. Factors that increase the duration of protection include reducing the frequency of pre-existing resistant alleles, the probability of nonrecombinational DNA repair, the probability of homing-associated loss of the effector, the fitness costs of the nuclease and effector, and the completeness of parasite blocking. For target species that extend over an area much larger than the typical dispersal distance, the duration of protection is expected to be highest at the release site, and decrease away from there, eventually falling to zero, as effector-less drive constructs replace effector-containing ones. We also model an alternative strategy of using the nuclease to target an essential gene, and then linking the effector to a sequence that restores the essential function and is resistant to the nuclease. Depending upon parameter values, this approach can prolong the duration of protection. Our models highlight the key design criteria needed to achieve a desired level of public health benefit.

MANY human diseases are transmitted indirectly, by vectors such as mosquitoes (*e.g.*, malaria, dengue fever, yellow fever, lymphatic filariasis), sandflies (leishmaniasis), tsetse flies (African trypanosomiasis), black flies (onchocerciasis), ticks (Lyme disease, relapsing fever), and many others. The ongoing burden of disease—estimated at more than a billion cases, and a million deaths every year (World Health Organization 2017)—indicates an ongoing need for more effective interventions. One approach that has been much discussed, at least in the context of malaria and dengue control, is to genetically engineer the vectors to contain one or more novel “effector” genes that block the development of the pathogen, and then use the process of gene drive to spread those effectors through the vector population (Burt 2014; Champer *et al.* 2016). In the context of malaria, effectors that have been shown to, at least partially, inhibit transmission include antimicrobial peptides, single-chain antibodies, immune system activators, and peptides that bind to mosquito proteins (putative parasite receptors) in the midgut or salivary glands (Wang and Jacobs-Lorena 2013; Adelman *et al.* 2016).

Gene drive is a natural process of preferential inheritance that allows transposable elements, gamete killers, B chromosomes, homing endonuclease genes, and many other types of genetic elements to spread through populations over successive generations, even if they cause some harm to the host organism (Burt and Trivers 2006). Many different approaches have been proposed for making synthetic gene drive constructs able to spread effector genes through a vector population (Marshall and Akbari 2016). One such approach uses genes encoding enzymes (nucleases) that recognize and cleave a specific DNA sequence (Burt 2003). If the gene is inserted in the middle of its own recognition sequence, thereby protecting the chromosome it is on from being cut, then it can catalyze the homing reaction, in which individuals that are heterozygous for the presence of the gene are converted to homozygotes, as the cut chromosome uses the intact one as a template for repair. The gene is then transmitted to all the progeny, rather than the Mendelian 50%, allowing it to increase rapidly in frequency in a population over successive generations. If an effector gene is linked to the nuclease gene, then it could also spread through the population. Proof-of-principle demonstrations of homing constructs carrying a cargo gene have been published in *Drosophila* and *Anopheles* using a natural homing endonuclease and engineered zinc finger, TALE, and CRISPR nucleases (Chan *et al.* 2011, 2013a,b; Simoni *et al.* 2014; Gantz and Bier 2015; Gantz *et al.* 2015; Hammond *et al.* 2016).

The homing reaction depends upon cleavage of the target DNA followed by recombinational repair using the construct-containing homologous chromosome as a template. This reaction is not 100% effective: for example, there are other pathways for repairing broken chromosomes, including nonhomologous end-joining and micro-homology mediated end-joining (Symington and Gautier 2011), and these other processes can produce “resistant” alleles that neither contain the nuclease gene nor are recognized by the nuclease. Moreover, mutations can occur in the construct while it is being copied during the homing reaction, and there is some evidence that the fidelity of copying at this stage may be lower than for normal DNA replication (Hicks *et al.* 2010; Simoni *et al.* 2014; Rodgers and McVey 2016). If mutations were to occur in the effector gene that rendered it nonfunctional, effectively “losing the cargo,” then it is possible a nuclease-only construct could spread through a population, with no effect on disease transmission.

Population modeling can be used to identify the key molecular and demographic parameters determining the fate of a gene drive construct after release, and its impact on pathogen transmission. It can therefore be useful both for designing constructs, and for determining whether laboratory results for a particular construct are sufficiently promising to consider proceeding to the field. Since the idea of using nucleases for population genetic engineering was first proposed (Burt 2003), there has been some modeling of simpler homing constructs designed to knock-out a target gene in the vector (Deredec *et al.* 2008, 2011; North *et al.* 2013; Bull 2017; Unckless *et al.* 2017), but less of constructs designed to knock-in a novel effector gene.

In this paper, we first model the simplest strategy of linking the effector directly to the nuclease, and explore how the duration of protection is affected by the probabilities of different molecular processes, by the fitness costs of the nuclease and the effector, and by the efficacy of the effector. We then explore the spatial dynamics, and find that the duration of protection is expected to decrease away from the release site, eventually to zero. Finally, we model an alternative strategy in which a nuclease is designed to target an essential gene, and the effector is linked to a sequence that restores the essential function, but is resistant to the nuclease (Burt 2003), and find that, in some circumstances, it can prolong the duration of protection. Our modeling will help guide the design and assessment of homing-based constructs for population-wide knock-in of novel transmission-blocking genes.

## Model I. Linking an effector to the nuclease

### Genetics

**Figure 1:**
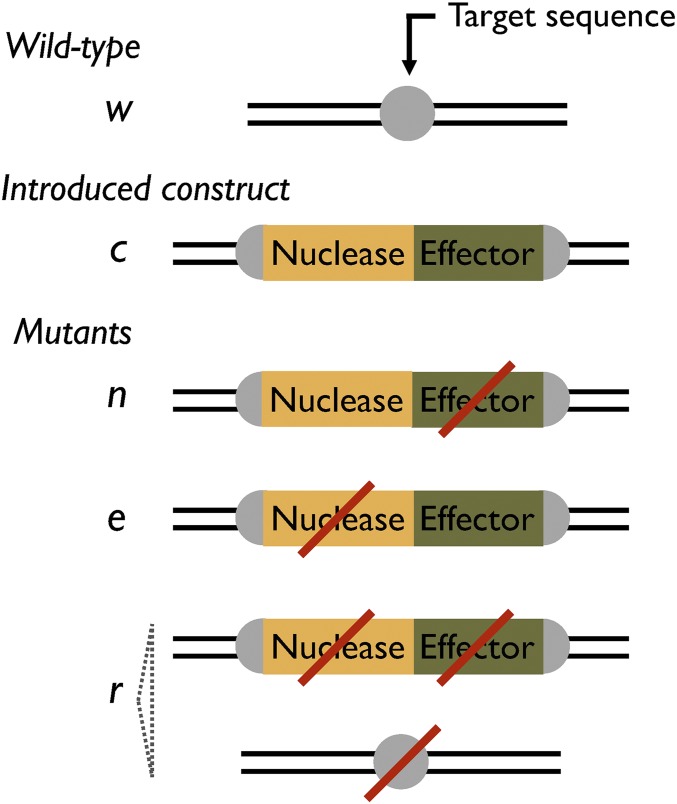
Five alleles in Model I. *w*, wild-type allele with sequence (typically 15–20 bp) recognized by the nuclease; *c*, complete construct that contains genes encoding the nuclease engineered to recognize the target sequence and the effector. In w/c heterozygotes, the nuclease is expressed in the germline and cleaves the target sequence, and the construct can “home” across to the cut chromosome during the repair process; the presence of the construct in the middle of its target sequence protects the *c* allele from being cut. *n*, nuclease-only constructs formed by loss of the effector gene during homing. *e*, effector-only construct formed by loss of the nuclease gene during homing. *r*, resistant allele that does not code for anything, and is not cut by the nuclease; they can be formed by loss of the genes during homing, or by non-HR of *w* alleles following cleavage.

The first model considers five alleles (Figure 1): the wild-type sequence (denoted *w*); the complete construct that has both an intact nuclease gene and an intact effector gene (*c*); nuclease-only constructs that have an intact nuclease gene, but defective effector (*n*); effector-only constructs that have an intact effector gene, but defective nuclease (*e*); and resistant alleles that are not recognized by the nuclease, and do not have either an intact nuclease gene or effector (*r*). We assume the population starts predominantly with the wild-type allele, and a certain number of individuals homozygous for complete constructs are introduced into it. Nuclease-only and effector-only alleles arise due to mutations during homologous repair (HR) events, while resistant alleles can arise in multiple ways: they may pre-exist in the population before release (due to sequence polymorphisms at the target site); they may arise from non-HR events after nuclease-induced cleavage (*e.g.*, nonhomologous end-joining or micro-homology-mediated repair); or they may arise due to mutations during HR events. Resistance alleles may therefore differ substantially in their primary sequence (as *n* and *e* alleles may also differ in sequence depending on the underlying mutation), but we shall consider them all to have the same transmission rates and effects on fitness.

Cleavage and homing can occur in the germlines of two genotypes, *w/c* and *w/n*. In *w/c* heterozygotes, we suppose cleavage of the target site occurs with probability $k_{c},$ and therefore the *w* allele is left uncleaved with probability $1-k_{c};$ cleaved alleles are repaired by non-HR (producing an *r* allele) with probability *k_j_*, and by HR with probability $1-k_{j};$ and HR leads to a mutation that disrupts the nuclease gene only (producing an *e* allele) with probability *k_n_*, a mutation that disrupts the effector gene only (producing an *n* allele) with probability *k_e_*, a mutation that disrupts both genes (producing an *r* allele) with probability $k_{ne},$ or no mutation in either gene (producing a *c* allele) with probability $1-k_{n}-k_{e}-k_{ne}.$ In *w/n* heterozygotes, we assume all probabilities are the same as above, except if cleavage is followed by HR; then there are only two options: a mutation disrupts the nuclease gene (producing an *r* allele, probability *k_n_*), or there is no mutation, leading to an *n* allele (probability $1-k_{n}$). In all other individuals, transmission is assumed to be Mendelian. The frequencies of each allele in the gametes of each genotype are shown in Supplemental Material, Table S1.

### Population biology

We assume the vector population consists of equal numbers of males and females, and that all genetic and fitness parameters are the same between them, so allele and genotype frequencies will be the same in the two sexes. For simplicity, we further assume that adults give rise to adults directly, without modeling the juvenile stages (eggs, larvae, pupae). New adults are recruited (born) according to a logistic density-dependent rate, and die at a constant rate. Each newly recruited adult is formed from a random mating event immediately previously [*i.e.*, we ignore sperm retention—see Beaghton *et al.* (2016)].

With five alleles, there are 15 different diploid genotypes, and their fitnesses are denoted relative to the wildtype homozygote (*w/w*), which has a fitness of one. Rather than have 14 different parameters for these fitnesses, we model them as functions of just six parameters: $s_{d},$ $s_{n}$ and $s_{e}$ are the homozygous fitness costs of disrupting the target sequence ($s_{d}$), of expressing the nuclease ($s_{n}$), and of expressing the effector ($s_{e}$), and $h_{d},$ $h_{n},$ $h_{e}$ are the corresponding dominance coefficients. $s_{d},$ $s_{n}$ and $s_{e}$ range from 0 (no cost) to 1 (lethal), and $h_{d},$ $h_{n},$ $h_{e},$ from 0 (completely recessive cost) to 1 (completely dominant). Each genotype has a fitness value associated with how many of its target sites are disrupted, how many nuclease genes it is expressing, and how many effector genes it is expressing (0, 1, or 2 in each case), and the overall fitness of the genotype is calculated as the product of these three values (Table S1). Differences in fitness are manifest as differences in the contribution of each genotype to the overall recruitment rate. We further assume the population is large enough that stochastic effects can be ignored, and the dynamics of allele frequencies and genotype abundances can be modeled using deterministic differential equations. The system of 15 equations is given in File S1, and is solved numerically using Wolfram Mathematica (Wolfram Research, Inc. 2015).

The effect of a release on disease transmission will depend on the abundances of the different genotypes and on the reduction in vector competence when the effector is present in one (heterozygote) or two (homozygote) copies (*$h_{rc}$r_c_* and *r_c_*, respectively; again, the degree of refractoriness *r_c_* ranges from 0 [no effect] to 1 [complete blockage], and the dominance coefficient for refractoriness *h${}_{rc}$* varies from 0 [recessive] to 1 [dominant]). We quantify the overall effect of the intervention at time *t* as the proportionate reduction in the vectorial capacity: Λ[t] = 1 − $V\left[t\right]/V_{0},$ where V[t] is the vectorial capacity at time *t*, calculated as the sum of the numbers of the different genotypes, weighted by their vector competence, and $V_{0}$ is the initial, pre-release vectorial capacity (see File S1 for further details). The spread of genes with fitness costs can reduce the total density of females, which will contribute to the reduction in vectorial capacity, but for most of the parameter values we consider in this paper the vast majority of the effect is through the increased proportion of individuals carrying the effector.

## Results

**Figure 2:**
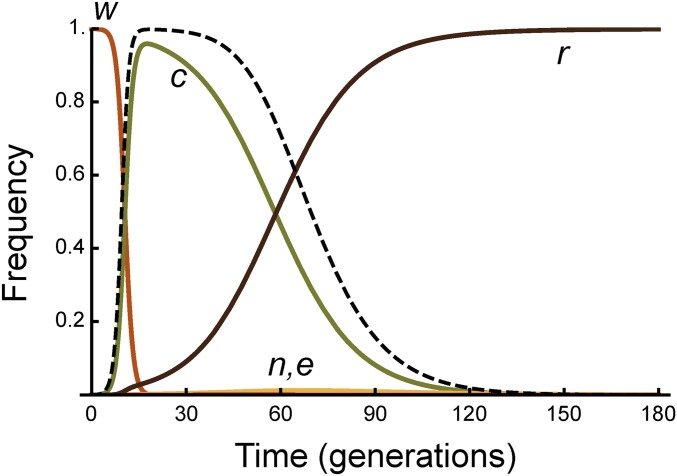
Allele frequency dynamics in Model I for the baseline set of parameter values. The complete construct (*c*) rapidly increases in frequency, replacing the wild type allele (*w*), but then is itself replaced by the resistant allele (*r*). The nuclease-only (*n*) and effector-only (*e*) alleles never reach appreciable frequencies with these parameter values. The dashed line shows Λ[t], the proportionate reduction in vectorial capacity.

There are 17 parameters in the model, defining the various aspects of demography, molecular biology, fitness and vector competence effects, and initial allele frequencies (Table 1). These are expected to vary according to the target species and molecular construct. To gain insight into the model, we have chosen an exemplar set of parameter values that is consistent with the most extensive published work on mosquitoes (Hammond *et al.* 2016), hypothetical homozygous fitness costs for nuclease expression of 5% and effector expression of 10%, with dominance coefficients for both of 0.5, and an effector that completely blocks transmission even in the heterozygous state (see Table 1 for other baseline parameter values). We then vary each of the parameters individually, while keeping the others at their baseline values, to determine which parameters are the most important in affecting the efficacy of the intervention. The homing rates in our baseline model are high [$k_{c}\left(1-k_{j}\right)\left(1-k_{n}-k_{e}-k_{ne}\right)$ = 97.5%], and, therefore, the construct increases rapidly in the population, replacing the wild type (Figure 2). However, resistant alleles are produced after $k_{j}+\left(1-k_{j}\right)k_{ne}=2.01\%$ of these initial cleavage events, and, because they do not suffer the costs of nuclease and effector expression, they gradually replace the complete constructs. Nuclease-only, or effector-only, alleles are produced after only 0.01% of cleavage events, and never attain a significant frequency. The proportionate reduction in vectorial capacity increases to a maximum of Λ[t] = 99.8%, and then falls back to 0. To quantify the duration of protection, we calculate the number of generations for which Λ[t] is >95 and 67%—and for our baseline parameter values this is 30 and 52 generations, respectively. To put these numbers into context, *Anopheles gambiae* mosquitoes may have 10–18 generations per year, depending on temperature (Depinay *et al.* 2004; Mordecai *et al.* 2013).

**Table 1:** Parameters and baseline values

Symbol	Parameter*^a^*	Baseline Value*^b^*
*λ*	Density-independent recruitment rate	6
*γ*	Density-dependent recruitment rate	1
$r_{0}$	Initial frequency of resistance	0
$cc_{0},wn_{0},ee_{0}$	Initial release frequencies	$10^{-4}$
$k_{c}$	Probability of cleavage	0.995
$k_{j}$	Probability of non-HR	0.02
$k_{n}$	Probability nuclease gene lost during homing	$10^{-4}$
$k_{e}$	Probability effector lost during homing (I)	$10^{-4}$
$k_{ne}$	Probability nuclease and effector lost during homing (I)	$10^{-4}$
$k_{r}$	Probability that non-HR of *w* produces functional *r* allele (II)	0
$h_{d}$	Dominance coefficient for target site disruption	0.5/0.03
$s_{d}$	Cost of target site disruption	0/1
$h_{n}$	Dominance coefficient for nuclease expression	0.5
$s_{n}$	Cost of nuclease expression	0.05
$h_{e}$	Dominance coefficient for effector expression	0.5
$s_{e}$	Cost of effector expression	0.1
$h_{rc}$	Dominance coefficient for refractoriness	1
$r_{c}$	Homozygous degree of refractoriness	1
$m_{w}$	Probability of spontaneous mutation of wild type allele (II)	$10^{-5}$
$m_{n}$	Probability of spontaneous mutation of nuclease allele (II)	$10^{-5}$
$m_{e}$	Probability of spontaneous mutation of effector allele (II)	$10^{-5}$

a I, Model I only; II, Model II only.

b Given as Model I/Model II if different.

**Figure 3:**
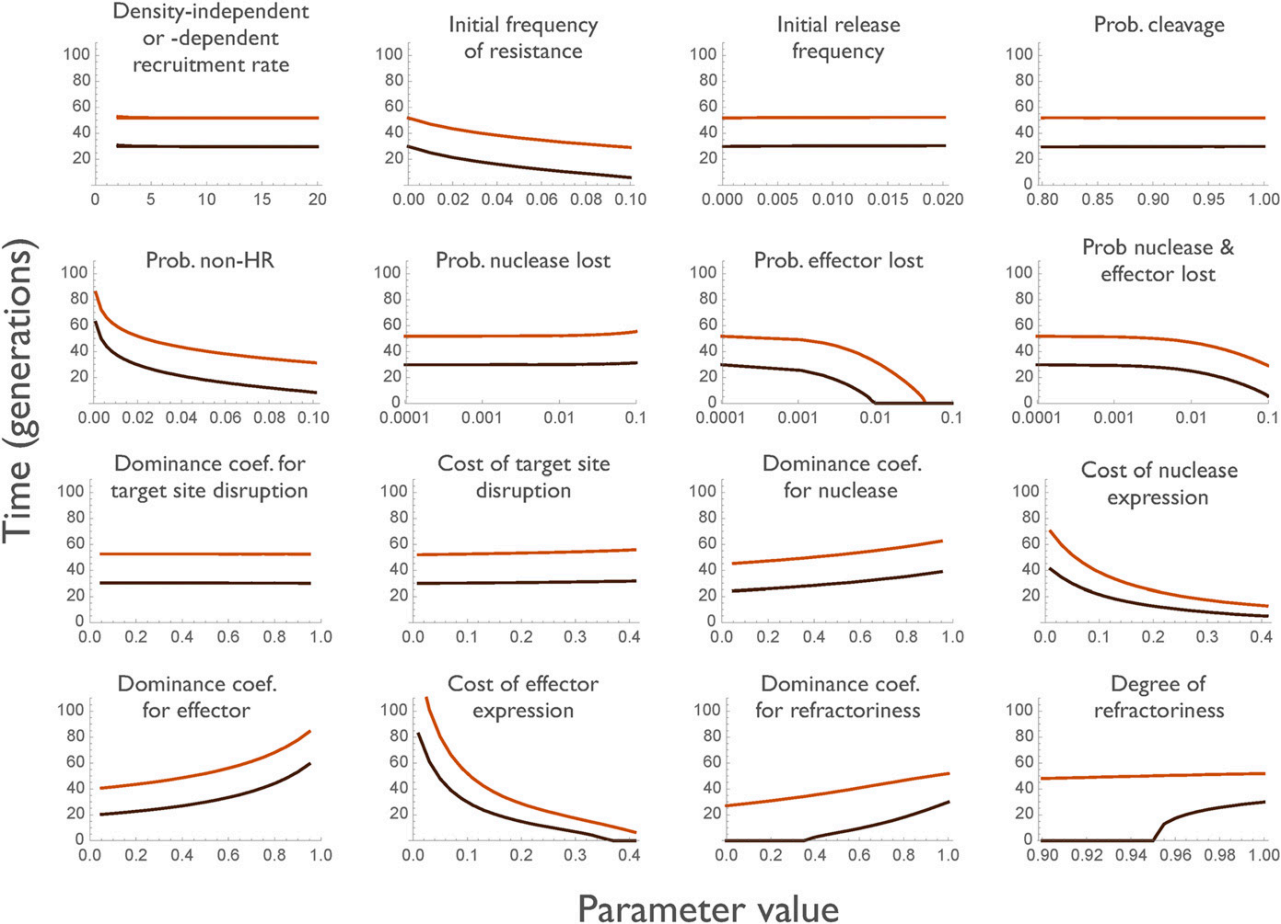
Duration of 67 and 95% protection (top and bottom lines in each graph) as each of the underlying parameters is varied, holding all others at their baseline values. When varying $h_{d},$ $s_{d}$ was set equal to 0.1.

To see which parameters are most important in determining this duration of impact, we varied each one over what seemed a reasonable range, while keeping the other parameters fixed at their baseline values (Figure 3). A number of parameters have little or no effect on the duration of efficacy, including the birth rate parameters (*λ* and *γ*, because we are monitoring the *proportion* of individuals with one or two effectors, rather than the total number); the initial release frequency of the construct (at least up to 1%); the cleavage rate (at least down to *k_c_* = 80%); the probability of homing-induced loss of the nuclease (*k_n_*); and the selection coefficients associated with disruption of the target locus (*h_d_* and *s_d_*, as long as they are not so high that the construct cannot spread). This last result follows from the fact that all the nonwild-type sequences suffer this cost, and so it does not affect the rate at which the effector-less alleles replace the effector-containing alleles. Factors that reduce the duration of protection include increasing (i) the frequency of pre-existing resistant alleles (*r${}_{0}$*), (ii) the probability of non-HR (*k_j_*), (iii) the probability of homing-associated loss of the effector (*k_e_*); (iv) the selection coefficient against the nuclease (*s_n_*); and (v) the selection coefficient against the effector (*s_e_*); and decreasing (vi) the completeness of blockage by the effector (*r_c_* and *h${}_{rc}$*). Interestingly, the more dominant the costs of nuclease and effector expression, the longer the duration of protection, because then the selective benefit of the resistant allele is recessive, and selection for rare recessives is slow. It is also possible to investigate how simultaneous variation in multiple parameters affects the duration of protection. As an example, Figure S1 shows contour plots for the duration of 67 or 95% reductions in vectorial capacity as a function of the homozygous fitness cost of the nuclease and of the effector (assumed to be equal, $s_{e}=s_{n}=s$) and the rate of non-HR ($k_{j}$).

File S2 is a Wolfram CDF document that allows users to define their own parameter values and visualize the resulting allele frequency dynamics and duration of protection (CDF player is available for free download from Wolfram; Wolfram Research Inc. 2017). While, for many parameter values, the dynamics are qualitatively similar to those shown in Figure 3, it is also possible to get more complex behavior, including apparent cycles (Figure S2).

### Spatial analysis

The *c* (complete) and *n* (nuclease-only) alleles in our model can spread only in the presence of the *w* (wild type) allele. The *n* allele has homing rate equal to the *c* allele, and imposes less of a fitness cost, and therefore the ratio of *n*:*c* alleles increases monotonically with time. However, with the baseline parameter values, the *n* allele reaches a maximum frequency of only 1.3% because it is not present at the time of release; it is formed relatively rarely; and the *c* allele consumes the *w* alleles so quickly that there is not enough time for *n* to significantly replace *c* before the resource upon which they depend (*w*) is exhausted and they both disappear, replaced by *r*. This model considered a single well-mixed population; in a spatial model with local dispersal and a single release site, the genes may spread out spatially from the release site, which would extend the competition between *n* and *c* alleles. One might therefore expect that *n* alleles will become more prominent away from the release site, and the intervention less effective.

To investigate this effect quantitatively, we formulate a system of reaction-diffusion equations for genotype dynamics that extends the model to two spatial dimensions (Fisher 1937; Shigesada and Kawasaki 1997; Beaghton *et al.* 2016). The main additional assumptions of the model are that organisms move randomly and are distributed continuously across a homogeneous environment much larger than the typical dispersal distance. Further details are given in File S1.

**Figure 4:**
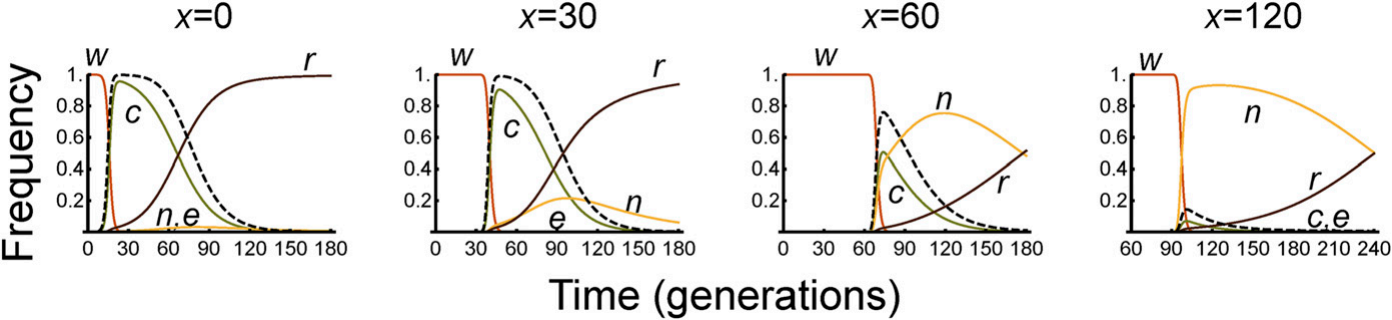
Allele frequency dynamics at different distances (*x*) from the release site in the spatial version of Model I. Distances are measured in units of the average movement per generation. The dashed line shows Λ, the proportionate reduction in vectorial capacity. Note time axis is shifted in the last panel.

**Figure 5:**
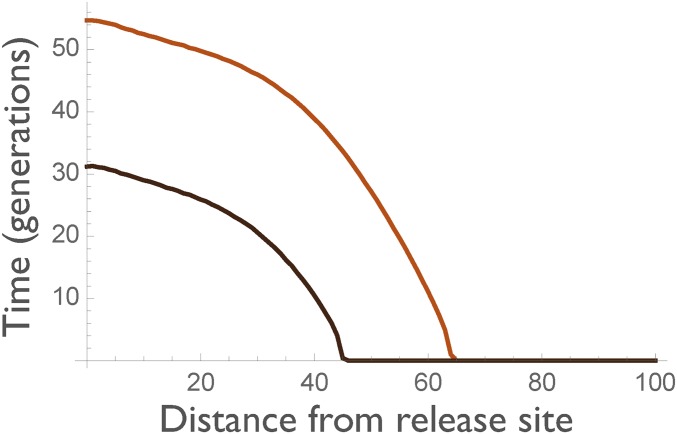
Duration of 67% (top line) and 95% (bottom) protection as a function of distance from the release site.

Analysis of the model shows that, as expected, it takes time for constructs released at one site to reach and have an effect at another site (Figure 4). Moreover, the *n* allele becomes (transiently) more prominent away from the release site, replacing the *c* allele, before itself being replaced by the *r* allele. As a result, the *c* allele reaches a lower maximum frequency, and the duration of protection is reduced (Figure 5). At sufficiently great distances, the effector never reaches an appreciable frequency, and the intervention has no protective effect.

## Model II. Linking the effector to a resistant gene

### Genetics

**Figure 6:**
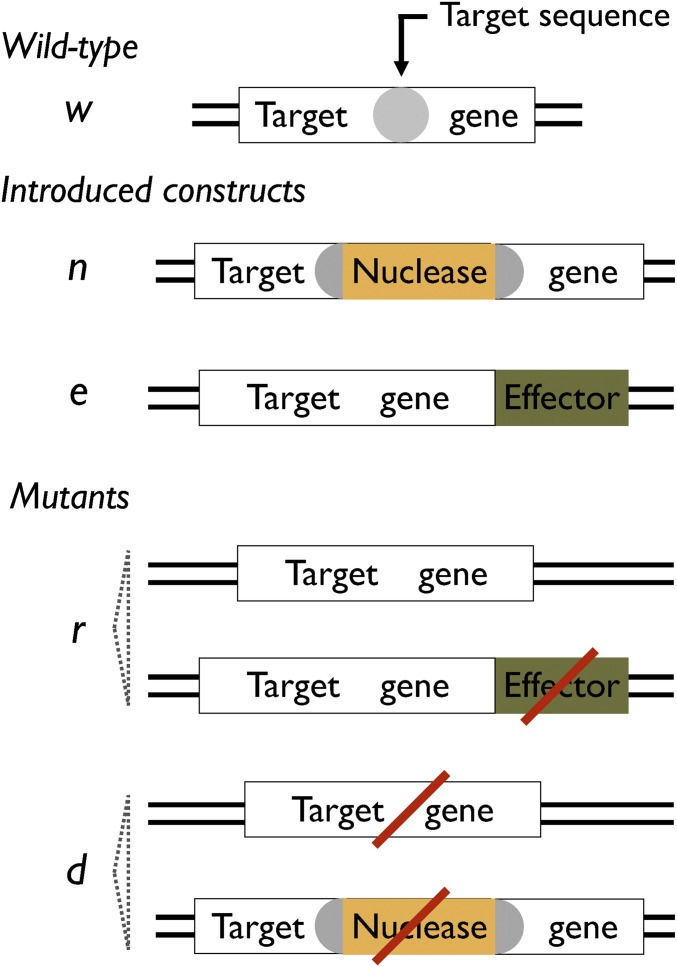
Five alleles in Model II. *w*, wild-type allele with target genes containing sequence recognized by the nuclease. *n*, allele with nuclease gene inserted in the middle of the target sequence, protecting the chromosome from being cut but also disrupting the target gene. *e*, effector gene linked to a target gene in which the recognition sequence has been changed so it is no longer recognized by the nuclease. *d*, disrupted target gene formed by non-HR of *w* alleles or by loss of nuclease from *n* alleles. *r*, functional target gene that is also resistant to cleavage due to not having the target sequence; can be formed by non-HR of *w* alleles or by loss of the effector gene of *e* alleles. Note that other alleles are possible, such as effector with disrupted target gene (*e.g.*, formed by spontaneous mutation of *e* alleles), or effector with functional target gene with target sequence (*e.g.*, formed by recombination between *w* and *e* alleles). These are expected to be rare because they are formed rarely and are not selected for.

As we have seen, if the effector is linked to the nuclease, the complete construct can spread rapidly, but it is then susceptible to being replaced by resistant alleles. The speed with which this second replacement occurs (and thus the speed with which protection is lost) depends upon the rate at which *r* alleles appear and their fitness advantage relative to *c* alleles. *r* alleles can appear relatively frequently (by non-HR), and their fitness advantage is larger because the effector is linked to the nuclease, which may have its own costs. An alternative approach that addresses these issues is to release a homing construct that targets a gene important for the host (*e.g.*, an essential gene), and a separate construct that is resistant to the nuclease, functional for the host, and has the effector linked to it (Figure 6). In principle, the advantages of this approach are that the effector-less functional resistant sequence will arise at a lower rate than in the previous model, and will be less strongly selected, which together should prolong the protection. For this approach to work, the knock-out phenotype (*e.g.*, death) must be recessive; non-HR events must not typically produce resistant alleles that are functional for the host; and yet it still must be possible to create a resistant sequence in the laboratory that is functional.

To quantify the duration of protection with this alternative approach, we constructed another model, again with five alleles: wildtype (*w*), nuclease only (*n*), effector with functional resistance (*e*), defective alleles that are resistant to cleavage but non-functional (*d*), and functional resistant alleles (*r*). For simplicity, we assume the *r* allele is fully functional, with no fitness cost. The population is assumed to start predominantly with *w* alleles, and a relatively small number of *n* heterozygotes and *e* homozygotes are introduced into it. Again, there are 15 different diploid genotypes. Homing can only occur in *w/n* heterozygotes, and is governed by the same processes as before, with parameters *k_c_*, *k_j_*, and *k_n_*, except for those gametes formed by non-HR: we let a fraction *k_r_* of them be functional resistant *r* alleles, with the remainder being defective *d* alleles (probability 1 − *k_r_*). We also now allow for loss of function of the nuclease, effector and target genes by spontaneous mutation (*i.e.*, not associated with homing), with baseline values for each of these set at 10${}^{-5}$ (Table 1). Such mutations are assumed to occur in the germline before homing. For simplicity, we continue assuming the target population is large enough that we do not have to worry about stochastic effects. Further details of the model are given in File S1, Table S2, Table S3 and Table S4.

## Results

**Figure 7:**
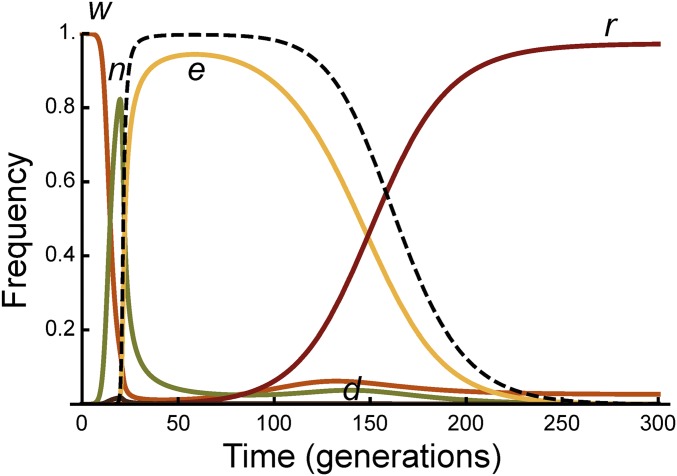
Allele frequency dynamics for Model II for the baseline set of parameter values. The nuclease allele (*n*) rapidly increases in frequency, replacing the wild type allele (*w*), but then is itself replaced by the containing allele (*e*), which in turn is eventually replaced by the resistant allele (*r*). The disrupted allele (*d*) never reaches an appreciable frequency with these parameter values. The dashed line shows Λ[t], the proportionate reduction in vectorial capacity.

**Figure 8:**
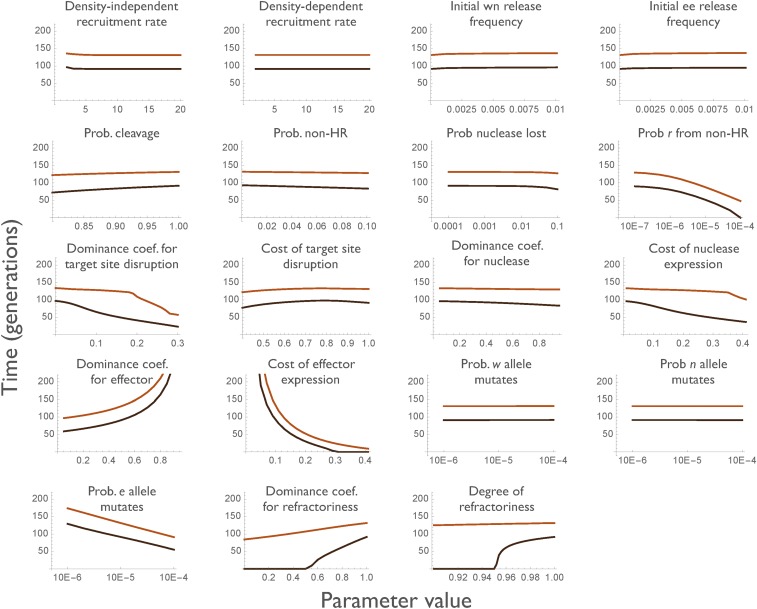
Duration of 67 and 95% protection (top and bottom lines in each graph) for Model II as each of the underlying parameters is varied, holding all others at their baseline values.

The trajectories of allele frequencies and the protection provided with the baseline parameter values are shown in Figure 7. The wildtype allele is rapidly replaced by the nuclease-only construct, which is then rapidly replaced by the resistant allele carrying the effector, which is then eventually replaced by the effector-less resistant allele. For these particular parameter values, the maximum level of protection is 99.7%, and protection remains above 95 and 67% for 90 and 129 generations, respectively. The effect of varying each parameter individually on the duration of protection is shown in Figure 8. As expected, in addition to the efficacy of the effector, the most important parameters are the fitness cost of the effector (*h_e_* and *s_e_*), and the probability an effector-less resistant allele arises either by non-HR (*k_r_*) or by spontaneous mutation (*m_e_*). File S3 includes an interactive Wolfram CDF document, allowing users to define their own parameter values and visualize the results. In general, the duration of protection is greater under Model II than under Model I, if the construct can be designed such that *r* alleles are sufficiently unlikely to arise from non-HR, but the extent of the advantage varies widely (*e.g.*, 20% to fourfold), depending on the assumptions made.

## Discussion

Drive can spread a gene through a population even if it is harmful to the organisms carrying it, but if a gene is both harmful and does not contribute to the drive, as with the effectors considered here, then loss-of-function mutations will eventually replace the functional gene. While an intervention may not be evolutionarily stable in the face of such mutations, the protection may nonetheless persist sufficiently long as to be worthwhile from a public health perspective. In this paper, we have presented a quantitative framework that indicates the design criteria needed to achieve a desired level of efficacy for two alternative approaches using the homing reaction to drive an effector gene through a vector population.

With both approaches, three types of parameters are important in determining the duration of protection: the probabilities of different molecular processes, the various fitness effects, and the efficacy of the effector in blocking transmission. Unfortunately, all of these may be challenging to measure, considering that performance in the field may be different than that in the laboratory [particularly for the effects on fitness and vector competence; Adelman *et al.* (2016)]. Field releases of effectors not associated with a drive system may be one source of useful information on these parameters.

Our modeling shows that the protection offered by the effector will be limited not only in time, but also in space, declining away from the release site. This is because, in the time taken for the construct to reach a distant site, there will have been more opportunity for effector-less constructs to replace effector-containing ones. At sufficient distances from the release site, only effector-less drive constructs are expected to spread through the population. The quantitative details of this effect will depend upon the spatial distribution and dispersal patterns of the target species. Unfortunately, for many vector species, these are poorly known; we have used a simple model of uniform distribution and random movement, but other possibilities should be considered.

The modeling presented here may be useful in designing gene drive constructs. For the simpler approach of linking the effector to the nuclease, the probability of non-HR is a key parameter to be minimized, and there is still much to learn about how best to do this. For example, different nuclease architectures leave different types of ends at the cleavage site, which may affect the repair pathway (Simoni *et al.* 2014). The choice of promoter used to drive nuclease expression will affect the cell type in which cleavage occurs, and can also affect the relative probabilities of different repair pathways (Chan *et al.* 2011, 2013a,b). Genomic context may also have an effect—for example, if there are repeats near the target site, this can stimulate micro-homology mediated repair (Nakade *et al.* 2014). With CRISPR-based nucleases, there is the option of using multiple gRNAs (Esvelt *et al.* 2014), and what effect this will have on the relative likelihood of HR needs investigating. It is also important to minimize the probability that homing leads to transfer of the nuclease gene but loss of the effector. For CRISPR-mediated drive, some incomplete HR events can occur if the gRNA locus is used as a template for repair (Hammond *et al.* 2016), and it may help to put the effector between the gRNA and Cas9 genes.

With the alternative approach of using the nuclease to target an essential gene, and linking the effector to a resistant allele, the probability of non-HR may be less important, but other parameters are critical, including targeting an important gene (*s_d_* high), where the knock-out is recessive (*h_d_* low), and it is possible to have a functional resistant allele, yet it is unlikely to arise by non-HR (*k_r_* low). In principle one could also combine the two approaches and link the effector both to the nuclease and to the resistant allele. Other variants are also possible, such as linking the effector to a *trans*-acting suppressor (*e.g.*, based on RNAi) or a mutator (*e.g.*, Wu *et al.* 2016) of the gene drive construct, either of which could be inserted elsewhere in the genome and could spread by natural selection. If no resistant, suppressor, or mutator allele was released, the nuclease will impose a load upon the population, which can lead to population suppression, or even elimination—yet another way to reduce disease transmission (Burt 2003; Deredec *et al.* 2008, 2011; Eckhoff *et al.* 2017). The possibility of eventually combining population suppression and population modification approaches should be considered. Yet other approaches to synthetic gene drive systems do not use the homing reaction, such as those mimicking MEDEA elements or based on underdominance (Marshall and Akbari 2016), and these will have their own design criteria for maximizing the duration of protection.

The models presented here could be extended in several directions. We have only considered the spread of a single effector gene, whereas it is possible that more than one will be needed for complete blockage (*e.g.*, Isaacs *et al.* 2012), and these could be in the same construct or in separate ones. It will also be useful to allow for sex-specific molecular and fitness parameters, as these may well differ between males and females (*e.g.*, if the effector is expressed only in females). We have also assumed that only the intended genotypes are released (*i.e.*, there is some way to prevent resistant genotypes from accumulating in the insectary). Our model also assumes an effectively infinite population, and allowing for some stochasticity may be particularly important for Model II, where our baseline rate for creation of functional resistant alleles is 10^−5^. In finite populations, there may be a nontrivial waiting time for such an allele to arise and survive stochastic loss, in which case protection would last longer than our current model indicates. We have also modeled the fitness cost of the effector as a constant, whereas if it acts early enough to block infection of the vector, and the pathogen is both common and harmful to the vector, then the effector may have low cost initially, and then increase in cost as the pathogen decreases in abundance. Finally, we have also treated the efficacy of the effector as a constant, and not included the possibility that the pathogen population might evolve in response to it. *Plasmodium*, for example, is a highly polymorphic pathogen, whose sequence diversity has been shown to be relevant to the partial protection seen in a recent vaccine trial (Neafsey *et al.* 2015). It will be important to assess any candidate effector gene against a diverse array of pathogen genotypes, and incorporate the results into models before release.

## Supplementary Material

Supplemental material is available online at www.genetics.org/lookup/suppl/doi:10.1534/genetics.116.197632/-/DC1.

## References

[bib1] Adelman Z. N. Basu S. Myles K. M., 2016 Engineering pathogen resistance in mosquitoes, pp. 277–304 in Genetic Control of Malaria and Dengue, edited by Adelman Z. N. Academic Press, London.

[bib2] Beaghton A. Beaghton P. J. Burt A., 2016 Gene drive through a landscape: reaction-diffusion models of population suppression and elimination by a sex ratio distorter. Theor. Popul. Biol. 108: 51–69. 26704073 10.1016/j.tpb.2015.11.005

[bib3] Bull J. J., 2017 OUP: lethal gene drive selects inbreeding. Evol. Med. Public Health 2017: 1–16. 10.1093/emph/eow030 PMC5226014 28013241

[bib4] Burt A., 2003 Site-specific selfish genes as tools for the control and genetic engineering of natural populations. Proc. Biol. Sci. 270: 921–928. 12803906 10.1098/rspb.2002.2319 PMC1691325

[bib5] Burt A., 2014 Heritable strategies for controlling insect vectors of disease. Phil. Trans. R. Soc. B 369: 20130432. 24821918 10.1098/rstb.2013.0432 PMC4024225

[bib6] Burt A. Trivers R., 2006 Genes in Conflict: The Biology of Selfish Genetic Elements. Belknap Press of Harvard University Press, Cambridge.

[bib7] Champer J. Buchman A. Akbari O. S., 2016 Cheating evolution: engineering gene drives to manipulate the fate of wild populations. Nat. Rev. Genet. 17: 146–159. 26875679 10.1038/nrg.2015.34

[bib8] Chan Y. S. Naujoks D. A. Huen D. S. Russell S., 2011 Insect population control by homing endonuclease-based gene drive: an evaluation in Drosophila melanogaster. Genetics 188: 33–44. 21368273 10.1534/genetics.111.127506 PMC3120159

[bib9] Chan Y. S. Huen D. S. Glauert R. Whiteway E. Russell S., 2013 a Optimising homing endonuclease gene drive performance in a semi-refractory species: the Drosophila melanogaster experience. PLoS One 8(1): e54130. 23349805 10.1371/journal.pone.0054130 PMC3548849

[bib10] Chan Y. S. Takeuchi R. Jarjour J. Huen D. S. Stoddard B. L., 2013 b The design and in vivo evaluation of engineered I-OnuI-based enzymes for HEG gene drive. PLoS One 8(9): e74254. 24040217 10.1371/journal.pone.0074254 PMC3769382

[bib11] Depinay J.-M. O. Mbogo C. M. Killeen G. Knols B. Beier J., 2004 A simulation model of African anopheles ecology and population dynamics for the analysis of malaria transmission. Malar. J. 3: 29. 15285781 10.1186/1475-2875-3-29 PMC514565

[bib12] Deredec A. Burt A. Godfray H. C. J., 2008 The population genetics of using homing endonuclease genes in vector and pest management. Genetics 179: 2013–2026. 18660532 10.1534/genetics.108.089037 PMC2516076

[bib13] Deredec A. Godfray H. C. J. Burt A., 2011 Requirements for effective malaria control with homing endonuclease genes. Proc. Natl. Acad. Sci. USA 108: E874–E880. 21976487 10.1073/pnas.1110717108 PMC3203790

[bib14] Eckhoff P. A. Wenger E. A. Godfray H. C. J. Burt A., 2017 Impact of mosquito gene drive on malaria elimination in a computational model with explicit spatial and temporal dynamics. Proc. Natl. Acad. Sci. USA 114: E255–E264. 28028208 10.1073/pnas.1611064114 PMC5240713

[bib15] Esvelt K. M. Smidler A. L. Catteruccia F. Church G. M., 2014 Concerning RNA-guided gene drives for the alteration of wild populations. eLife 3: e03401. 25035423 10.7554/eLife.03401 PMC4117217

[bib16] Fisher R. A., 1937 The wave of advance of advantageous genes. Ann. Eugen. 7: 355–369.

[bib17] Gantz V. M. Bier E., 2015 The mutagenic chain reaction: a method for converting heterozygous to homozygous mutations. Science 348: 442–444. 25908821 10.1126/science.aaa5945 PMC4687737

[bib18] Gantz V. M. Jasinskiene N. Tatarenkova O. Fazekas A. Macias V. M., 2015 Highly efficient Cas9-mediated gene drive for population modification of the malaria vector mosquito *Anopheles stephensi*. Proc. Natl. Acad. Sci. USA 112: E6736–E6743. 26598698 10.1073/pnas.1521077112 PMC4679060

[bib19] Hammond A. Galizi R. Kyrou K. Simoni A. Siniscalchi C., 2016 A CRISPR-Cas9 gene drive system-targeting female reproduction in the malaria mosquito vector *Anopheles gambiae*. Nat. Biotechnol. 34: 78–83. 26641531 10.1038/nbt.3439 PMC4913862

[bib20] Hicks W. M. Kim M. Haber J. E., 2010 Increased mutagenesis and unique mutation signature associated with mitotic gene conversion. Science 329: 82–85. 20595613 10.1126/science.1191125 PMC4254764

[bib21] Isaacs A. T. Jasinskiene N. Tretiakov M. Thiery I. Zettor A., 2012 Transgenic *Anopheles stephensi* coexpressing single-chain antibodies resist *Plasmodium falciparum* development. Proc. Natl. Acad. Sci. USA 109: E1922–E1930. 22689959 10.1073/pnas.1207738109 PMC3396534

[bib22] Marshall J. M. Akbari O. S., 2016 Gene drive strategies for population replacement, pp. 169–200 in Genetic Control of Malaria and Dengue, edited by Adelman Z. N. Academic Press, London.

[bib23] Mordecai E. A. Paaijmans K. P. Johnson L. R. Balzer C. Ben-Horin T., 2013 Optimal temperature for malaria transmission is dramatically lower than previously predicted. Ecol. Lett. 16: 22–30. 23050931 10.1111/ele.12015

[bib24] Nakade S. Tsubota T. Sakane Y. Kume S. Sakamoto N., 2014 Microhomology-mediated end-joining-dependent integration of donor DNA in cells and animals using TALENs and CRISPR/cas9. Nat. Commun. 5: 5560. 25410609 10.1038/ncomms6560 PMC4263139

[bib25] Neafsey D. E. Juraska M. Bedford T. Benkeser D. Valim C., 2015 Genetic diversity and protective efficacy of the RTS,S/AS01 malaria vaccine. N. Engl. J. Med. 373: 2025–2037. 26488565 10.1056/NEJMoa1505819 PMC4762279

[bib26] North A. Burt A. Godfray H. C. J., 2013 Modelling the spatial spread of a homing endonuclease gene in a mosquito population. J. Appl. Ecol. 50: 1216–1225. 25558082 10.1111/1365-2664.12133 PMC4277857

[bib27] Rodgers K. McVey M., 2016 Error-prone repair of DNA double-strand breaks. J. Cell. Physiol. 231: 15–24. 26033759 10.1002/jcp.25053 PMC4586358

[bib28] Shigesada N. Kawasaki K., 1997 Biological Invasions: Theory and Practice. Oxford University Press, Oxford.

[bib29] Simoni A. Siniscalchi C. Chan Y. Huen D. Russell S., 2014 Development of synthetic selfish elements based on modular nucleases in *Drosophila melanogaster*. Nucleic Acids Res. 42: 7461–7472. 24803674 10.1093/nar/gku387 PMC4066794

[bib30] Symington L. S. Gautier J., 2011 Double-strand break end resection and repair pathway choice. Annu. Rev. Genet. 45: 247–271. 21910633 10.1146/annurev-genet-110410-132435

[bib31] Unckless R. L. Clark A. G. Messer P. W., 2017 Evolution of resistance against CRISPR/cas9 gene drive. Genetics 205: 827–841. 27941126 10.1534/genetics.116.197285 PMC5289854

[bib32] Wang S. B. Jacobs-Lorena M., 2013 Genetic approaches to interfere with malaria transmission by vector mosquitoes. Trends Biotechnol. 31: 185–193. 23395485 10.1016/j.tibtech.2013.01.001 PMC3593784

[bib33] Wolfram Research, Inc., 2015 Mathematica 10.2. Wolfram Research, Inc., Hanborough, UK.

[bib34] Wolfram Research, Inc., 2017 Wolfram CDF Player. Available at: http://www.wolfram.com/cdf-player/. Accessed: January 9, 2017.

[bib35] World Health Organization. 2017 Vector-borne diseases. Available at: http://www.who.int/mediacentre/factsheets/fs387/en/. Accessed: January 9, 2017.

[bib36] Wu B. Luo L. Gao X. J., 2016 Cas9-triggered chain ablation of cas9 as a gene drive brake. Nat. Biotechnol. 34: 137–138. 26849513 10.1038/nbt.3444 PMC5326742

