# Supplementary material for: Requirements for Driving Antipathogen Effector Genes into Populations of Disease Vectors by Homing
Source: Genetics. 2017 Feb 2;205(4):1587–96. doi: 10.1534/genetics.116.197632 (PMC5378115; doi:10.1534/genetics.116.197632)
Supplement: Supplementary file 1 [file 1587FileS1.pdf]

File S1. Description of models.

We develop a system of continuous-time differential equations for genotype abundances. This approach allows us to conveniently include dispersal of genotypes (see below, Model I, Spatial analysis) and to incorporate effects of changing population size along with genetic selection (i.e., no assumptions made of constant or infinite population size). As noted in the text, we assume a 50:50 sex ratio, and that all genetic and fitness parameters are the same in males and females, so allele and genotype frequencies will also be the same. We further assume that adults give rise to adults directly: new adults are recruited (born) according to a logistic density-dependent rate, and die at a constant rate. The number of individuals (females and males) of genotype i/j is denoted by a_{ij}, where i and j refer to the allele types. There are 5 alleles (and therefore 15 genotypes) in each model (w, c, n, e, r for Model I and w, n, e, r, d for Model II). The total population size is $n(t) = \sum_{i=1}^{5} \sum_{j=i}^{5} a_{ij}$.

Fitness and selection

Let $w_{ij} \leq 1$ represent the fitness of genotype i/j relative to $w_{ww} = 1$ for the wild-type homozygote. Fitness effects are manifest as differences in the relative ability of of genotypes to participate in mating and reproduction. Effectively, only a reduced number of individuals of genotype i/j, given by $w_{ij}a_{ij}$, participate in reproduction and contribute gametes at time t. The total number of adults in the population that participate in reproduction at time t is therefore fitness-weighted:

$$\sum_{i=1}^{5} \sum_{j=i}^{5} w_{ij} a_{ij}(t) \tag{1}$$

The proportion $g_k(t)$ of type k alleles in the gametes produced by individuals participating in reproduction at time t is thus given by:

$$g_k(t) = \frac{\sum_{i=1}^{5} \sum_{j=i}^{5} c_{ij,k} w_{ij} a_{ij}(t)}{\sum_{i=1}^{5} \sum_{j=i}^{5} w_{ij} a_{ij}(t)} \tag{2}$$

The coefficients $c_{ij,k}$ in (2) specify the proportion of the gametes of i/j individuals that carry allele k, and include the results of cleavage and homing that occur during gamete formation (Model I) as well as mutation preceding those processes (Model II).

We may rewrite (2) for $g_k(t)$ as a function of the genotype frequencies $a_{ij}(t)/n(t)$ weighted by their normalized genotype fitness $w_{ij}/\bar{w}$:

$$g_k(t) = \sum_{i=1}^{5} \sum_{j=i}^{5} c_{ij,k} \frac{w_{ij}}{\bar{w}} \frac{a_{ij}(t)}{n(t)} \tag{3}$$

Above, the mean population fitness $\bar{w}$ is given by:

$$\bar{w} = \sum_{i=1}^{5}\sum_{j=i}^{5} w_{ij}[a_{ij}(t)/n(t)] \tag{4}$$

We further assume that the individuals that participate in mating choose partners randomly, and the genotypic composition of offspring is therefore equivalent to draws of pairs of gametes from the available gamete 'pool'.

Population biology

We use a logistic-type model with density-dependent birth rate and density-independent death rate. λ is the recruitment rate of the wild-type genotype at low density, γ is a parameter determining how the recruitment rate declines with density, and μ is the death rate. We impose logistic density dependence on the recruitment rates rather than the death rate to keep the average generation time ($1/\mu$) constant in the face of temporally variable population size. For simplicity, μ is assumed to be the same for all genotypes so that they all have the same generation time (one could, if desired, allow for decreased survivorship for less fit genotypes by assigning them an increased mortality rate compared to the wild-type, $\mu_{ij} > \mu_{ww}$). This leads to a differential equation for the total population size as a function of the individual genotype populations:

$$\frac{dn(t)}{dt} = \big(\lambda - \gamma n(t)\big)\sum_{i=1}^{5}\sum_{j=i}^{5} w_{ij}a_{ij}(t) - \mu n(t) = \big(\lambda - \gamma n(t)\big)\bar{w}n(t) - \mu n(t) \tag{5}$$

In this equation, the population-wide total recruitment rate at which new offspring are created is equal to the total number of individuals that participate in reproduction, $\bar{w}n(t)$ (consistent with our definition of fitness above) times the density-dependent recruitment rate per individual, $\lambda - \gamma n(t)$ (all individuals contribute equally to density-dependent competition, regardless of genotype). The dependence of the recruitment rate on fitness-weighted populations, $\bar{w}n(t) = \sum_{i=1}^{5}\sum_{j=i}^{5} w_{ij}a_{ij}(t)$, implies that individuals that participate in mating and reproduction are always able to find mates. Although here we do not differentiate between males and females, this model is consistent with the rate of new births being dependent on number and fitness of females, and the assumption that all participating females are fertilized (Beaghton et al 2016). It can be shown that if equations for male and female populations are derived separately using these assumptions, they reduce to (5) when the sex ratio is 50:50.

We now consider the continuous-time differential equations for the individual genotype populations. To find the total recruitment rate of individuals of genotype i/j, we multiply the total rate of production of all new offspring at time t by the fraction of births that result from a random mating between two (participating) individuals in which one contributed gamete i and the other j. This fraction is $2g_i(t)g_j(t)$ for $i \neq j$ and $g_i(t)^2$ for $i = j$, with $g_i(t)$ given by (3). Selection thus occurs through the relative participation of different genotypes in

mating and reproduction. This results in a nonlinear system of equations (15 in total) for the genotype abundances as the dependent variables, with $g_i(t)$ and $\bar{w}(t)$ given by (2) and (4), and $n(t) = \sum_{i=1}^{5} \sum_{j=i}^{5} a_{ij}$:

$$\frac{da_{ij}(t)}{dt} = \big(\lambda - \gamma n(t)\big)\bar{w}n(t)(2 - \delta_{ij})g_i(t)g_j(t) - \mu a_{ij}(t) \tag{6}$$

Here, δ_{ij} is the Kronecker delta, where $\delta_{ij} = 0$ if $i \neq j$ and 1 if $i = j$, and so the factor $(2 - \delta_{ij})$ accounts for the factor of two for heterozygotes and one for homozygotes. This set of differential equations may be solved numerically (Wolfram Mathematica). If we sum them up, as expected we obtain (5) for the rate of change of the total population $n(t)$.

For all analyses in Model I and II below, we set $\mu = 1$ so that time is measured in generations.

Model I

As described in the main text, there are five alleles (w, c, n, e, and r) and 15 diploid genotypes. The non-spatial equations (6) for the genotype abundances are:

$$\frac{da_{ww}(t)}{dt} = (\lambda - \gamma n(t))\bar{w}n(t)g_w(t)^2 - \mu a_{ww}(t)$$

$$\frac{da_{cc}(t)}{dt} = (\lambda - \gamma n(t))\bar{w}n(t)g_c(t)^2 - \mu a_{cc}(t)$$

$$\frac{da_{nn}(t)}{dt} = (\lambda - \gamma n(t))\bar{w}n(t)g_n(t)^2 - \mu a_{nn}(t)$$

$$\frac{da_{ee}(t)}{dt} = (\lambda - \gamma n(t))\bar{w}n(t))g_e(t)^2 - \mu a_{ee}(t)$$

$$\frac{da_{rr}(t)}{dt} = (\lambda - \gamma n(t))\bar{w}n(t)g_r(t)^2 - \mu a_{rr}(t)$$

$$\frac{da_{wc}(t)}{dt} = (\lambda - \gamma n(t))\bar{w}n(t)2g_w(t)g_c(t) - \mu a_{wc}(t)$$

$$\frac{da_{wn}(t)}{dt} = (\lambda - \gamma n(t))\bar{w}n(t)2g_w(t)g_n(t) - \mu a_{wn}(t)$$

$$\frac{da_{we}(t)}{dt} = (\lambda - \gamma n(t))\bar{w}n(t)2g_w(t)g_e(t) - \mu a_{we}(t)$$

$$\frac{da_{wr}(t)}{dt} = (\lambda - \gamma n(t))\bar{w}n(t)2g_w(t)g_r(t) - \mu a_{wr}(t)$$

$$\frac{da_{cn}(t)}{dt} = (\lambda - \gamma n(t))\bar{w}n(t)2g_c(t)g_n(t) - \mu a_{cn}(t)$$

$$\frac{da_{ce}(t)}{dt} = (\lambda - \gamma n(t))\bar{w}n(t)2g_c(t)g_e(t) - \mu a_{ce}(t)$$

$$\frac{da_{cr}(t)}{dt} = (\lambda - \gamma n(t))\bar{w}n(t)2g_c(t)g_r(t) - \mu a_{cr}(t)$$

$$\frac{da_{ne}(t)}{dt} = (\lambda - \gamma n(t))\bar{w}n(t)2g_n(t)g_e(t) - \mu a_{ne}(t)$$

$$\frac{da_{nr}(t)}{dt} = (\lambda - \gamma n(t))\bar{w}n(t)2g_n(t)g_r(t) - \mu a_{nr}(t)$$

$$\frac{da_{er}(t)}{dt} = (\lambda - \gamma n(t))\bar{w}n(t)2g_e(t)g_r(t) - \mu a_{er}(t)$$

Genotype fitnesses w_{ij} for Model I are given in the first column of Table S1. The (fitness-weighted) proportions of gamete types that are available for reproduction $\big(g_w(t), g_c(t), g_n(t), g_e(t)$, and $g_r(t)\big)$ are calculated using (2). The coefficients $c_{ij,k}$ in (2) that correspond to the proportion of the gametes of i/j individuals that carry allele k are shown in Table S1 (rows corresponding to genotype a_{ij} and columns to allele k).

Cleavage only occurs in w/c and w/n heterozygotes, and can result in perfect or inperfect homing. In all other genotypes, inheritance is Mendelian. We ignore spontaneous (i.e., not homing-related) loss-of-function mutations of nuclease and effector genes, or of w alleles to r. Similarly, we ignore the possibility of non-homing-associated recombination between n and e alleles producing either c or r alleles (note that e typically remains rare in the population), or between c and r alleles to produce n or e.

Initial conditions at the time of release ($t = 0$) are:

$$a_{ww}(0) = (1 - r_0)^2 n_0$$

$$a_{cc}(0) = cc_0\ n_0$$

$$a_{rr}(0) = (r_0)^2 n_0$$

$$a_{wr}(0) = 2r_0(1 - r_0)n_0$$

and all other genotype populations start at zero, where $n_0 = (\lambda - \mu)/\gamma$ is the equilibrium pre-release number of individuals, r_0 is the initial pre-release frequency of resistant alleles, and cc_0 is the number of released c/c homozygotes, as a proportion of the initial population n_0.

The proportionate reduction in vectorial capacity at time t, relative to the pre-intervention value, is:

$$\begin{aligned}\Lambda(t) &= 1 - V(t)/V_0 \\ &= 1 - \Big(a_{ww}(t) + a_{nn}(t) + a_{rr}(t) + a_{wn}(t) + a_{wr}(t) + a_{nr}(t) \\ &\quad + \big(a_{wc}(t) + a_{we}(t) + a_{cn}(t) + a_{cr}(t) + a_{ne}(t) + a_{er}(t)\big)(1 - h_{rc}r_c) \\ &\quad + \big(a_{cc}(t) + a_{ee}(t) + a_{ace}(t)\big)(1 - r_c)\Big)/n_0\end{aligned}$$

Note we define vectorial capacity here to be the expected number of new infections per day produced by a single infection, when infections are rare. More specifically, in the context of the classical Ross-McDonald model of malaria transmission (here in discrete time), vectorial capacity is equal to:

$$V = \frac{a^2\, b\, c\, \theta}{\mu} A$$

where A is the number of adult female mosquitoes per person; a is the daily probability a female feeds on a human; b is the probability an infectious mosquito biting a human transmits the parasite; c is the probability that a mosquito biting an infected person becomes infected; μ is the daily probability an adult female mosquito dies; and θ is the probability a female survives the period from being infected to becoming infectious (the extrinsic incubation period). Note that $\theta = (1-\mu)^T$, where T is the length of this period.

Thus vectorial capacity includes all the components of the intrinsic rate of increase of the disease (typically denoted R_0), except the recovery rate for humans (i.e., $R_0 = V/r$, where r is the daily probability an infected human clears the disease (or dies), and therefore $1/r$ is the average number of days a human remains infected (and infectious)). In principle, all components of V are susceptible to targeting by gene drive, whereas r is unlikely to be.

We define vector competence as V/A – that is, as including all the same terms as vectorial capacity, except the abundance of adult females. In our model, the efficacy of the effector is measured as the proportionate reduction in the competence of females that are heterozygous or homozygous for the effector (compared to wildtype). If the effector acts by reducing parameter b only, or c only, then the proportionate reduction in competence is equal to the proportionate reduction in these parameters, because competence is a linear function of b and c. If the effector acts to reduce the biting rate a, then what matters is the proportionate reduction in a^2. Similarly, if the effector acts to delay the development of the parasite, increasing T, then the proportionate reduction in competence will be equal to the proportionate reduction in θ. Finally, if the effector acts to increase the daily probability of an infected mosquito dying, then the proportionate reduction in competence will be equal to the proportionate reduction in θ/μ; note A may also be affected. For further details, see Brady *et al.* (2016).

Spatial analysis

For the spatial analysis, applied to Model I, we assume the target population is spread out uniformly across a uniform landscape and individuals move according to a random walk. For initial release of the construct in a circular area, the solution for the genotype densities is radially symmetrical, and we model the dynamics as a function of distance from the center of the release site, x. The density of genotype i/j at distance x from the release site at time t is denoted as $a_{ij}(x,t)$. The model now consists of a system of 15 partial differential equations that incorporate diffusion. For example, for genotype i/j, the equation becomes:

$$\frac{\partial a_{ij}(x,t)}{\partial t} = D\left(\frac{\partial^2 a_{ij}(x,t)}{\partial x^2} + \frac{1}{x}\frac{\partial a_{ij}(x,t)}{\partial x}\right)$$
$$+ \big(\lambda - \gamma n(x,t)\big)\bar{w}n(x,t)(2-\delta_{ij})g_i(x,t)g_j(x,t) - \mu a_{ij}(x,t) \tag{7}$$

where D is the diffusion coefficient, assumed to be the same for all genotypes, which quantifies the rate of movement of the organisms. For all analyses we measure distances in units of the average distance moved per generation (i.e., the average distance between where an individual and its parents or its offspring are born). Therefore, since time is measured in generations, $D = 1/\pi$ (Shigesada 1997).

We solve (7) on an unbounded domain, $0 \le x < \infty$. Initial conditions at the time of release ($t = 0$) are

$$a_{ww}(x,0) = n_0$$
$$a_{cc}(x,0) = \begin{cases} cc_0\ n_0, & 0 \le x \le 1 \\ 0, & x > 1 \end{cases}$$

with other genotype populations starting at zero everywhere.

Model II

As described in the main text, there are five alleles (w, n, e, r, and d). We assume the target, nuclease and effector genes are all subject to spontaneous mutations in the germline of individuals, before any possible cleavage or homing. As they occur in the germline, we assume these mutations do not affect the survival or reproduction of the individual in which they occur. The transition matrix showing the probabilities of each genotype mutating to each other is shown in Table S2.

Cleavage only occurs in w/n heterozygotes. Cleavage can result in perfect homing of the n allele, or imperfect homing that produces a d allele, or non-HR, resulting most of the time in a d allele, but occasionally in an r allele. In all other genotypes, inheritance is Mendelian. The relative proportion of each type of gamete produced by each genotype i/j due to homing and cleavage is shown in Table S3.

Shown in Table S4 are the coefficients $c_{ij,k}$ that represent the proportion of gametes of i/j individuals that carry allele k, incorporating both spontaneous mutation and cleavage followed by repair. Fitness w_{ij} of genotype i/j is shown in the first column of Table S4. As in Model I, these are used to calculate the (fitness-weighted) proportions of gamete types that are available for reproduction ($g_w(t), g_n(t), g_e(t), g_r(t)$, and $g_d(t)$). The differential equations for the genotype abundances for Model II are:

$$\frac{da_{ww}(t)}{dt} = (\lambda - \gamma n(t))\bar{w}n(t)g_w(t)^2 - \mu a_{ww}(t)$$

$$\frac{da_{nn}(t)}{dt} = (\lambda - \gamma n(t))\bar{w}n(t)g_n(t)^2 - \mu a_{nn}(t)$$

$$\frac{da_{ee}(t)}{dt} = (\lambda - \gamma n(t))\bar{w}n(t)g_e(t)^2 - \mu a_{ee}(t)$$
$$\frac{da_{rr}(t)}{dt} = (\lambda - \gamma n(t))\bar{w}n(t)g_r(t)^2 - \mu a_{rr}(t)$$
$$\frac{da_{dd}(t)}{dt} = (\lambda - \gamma n(t))\bar{w}n(t)g_d(t)^2 - \mu a_{dd}(t)$$
$$\frac{da_{wn}(t)}{dt} = (\lambda - \gamma n(t))\bar{w}n(t)2g_w(t)g_n(t) - \mu a_{wn}(t)$$
$$\frac{da_{we}(t)}{dt} = (\lambda - \gamma n(t))\bar{w}n(t)2g_w(t)g_e(t) - \mu a_{we}(t)$$
$$\frac{da_{wr}(t)}{dt} = (\lambda - \gamma n(t))\bar{w}n(t)]2g_w(t)g_r(t) - \mu a_{wr}(t)$$
$$\frac{da_{wd}(t)}{dt} = (\lambda - \gamma n(t))\bar{w}n(t)2g_w(t)g_d(t) - \mu a_{wd}(t)$$
$$\frac{da_{ne}(t)}{dt} = (\lambda - \gamma n(t))\bar{w}n(t)2g_n(t)g_e(t) - \mu a_{ne}(t)$$
$$\frac{da_{nr}(t)}{dt} = (\lambda - \gamma n(t))\bar{w}n(t)2g_n(t)g_r(t) - \mu a_{nr}(t)$$
$$\frac{da_{nd}(t)}{dt} = (\lambda - \gamma n(t))\bar{w}n(t)2g_n(t)g_d(t) - \mu a_{nd}(t)$$
$$\frac{da_{er}(t)}{dt} = (\lambda - \gamma n(t))\bar{w}n(t)2g_e(t)g_r(t) - \mu a_{er}(t)$$
$$\frac{da_{ed}(t)}{dt} = (\lambda - \gamma n(t))\bar{w}n(t)2g_e(t)g_d(t) - \mu a_{ed}(t)$$
$$\frac{da_{rd}(t)}{dt} = (\lambda - \gamma n(t))\bar{w}n(t)2g_r(t)g_d(t) - \mu a_{rd}(t)$$

The initial conditions at the time of release ($t = 0$) are:

$$a_{ww}\,[0] = n_0$$
$$a_{wn}\,[0] = wn_0\ n_0$$
$$a_{cc}\,[0] = ee_0\ n_0$$

where wn_0 is the number of released w/n heterozygotes and ee_0 is the number of released c/c homozygotes, both as a proportion of the initial population n_0. All other genotype populations start at zero.

The proportionate reduction in vectorial capacity at time t, relative to the pre-intervention value, is:

$$\Lambda(t) = 1 - V(t)/V_0$$
$$= 1 - \Big(a_{ww}(t) + a_{wn}(t) + a_{wr}(t) + a_{wd}(t) + a_{nn}(t) + a_{nr}(t) + a_{nd}(t) + a_{rr}(t) + a_{rd}(t) + a_{dd}(t)$$
$$+ \big(a_{we}(t) + a_{ne}(t) + a_{er}(t) + a_{ed}(t)\big)(1 - h_{rc}r_c)$$
$$+ a_{ee}(t)(1 - r_c)\Big)/n_0$$

References

Brady O.J., H. C. J. Godfray, A. J. Tatem, P. W. Gething, J. M. Cohen, F. E. McKenzie, T. A. Perkins, R. C. Reiner Jr, L. S. Tusting, M. E. Sinka, C. L. Moyes, P. A. Eckhoff, T. W. Scott, S. W. Lindsay, S. I. Hay and D. L. Smith, 2016 Vectorial capacity and vector control: reconsidering sensitivity to parameters for malaria elimination. Trans. R. Soc. Trop. Med. Hyg. **110**: 107-117. doi: 10.1093/trstmh/trv113.
