# Supplementary figures and images for: Requirements for Driving Antipathogen Effector Genes into Populations of Disease Vectors by Homing
Source: Genetics. 2017 Feb 2;205(4):1587–96. doi: 10.1534/genetics.116.197632 (PMC5378115; doi:10.1534/genetics.116.197632)

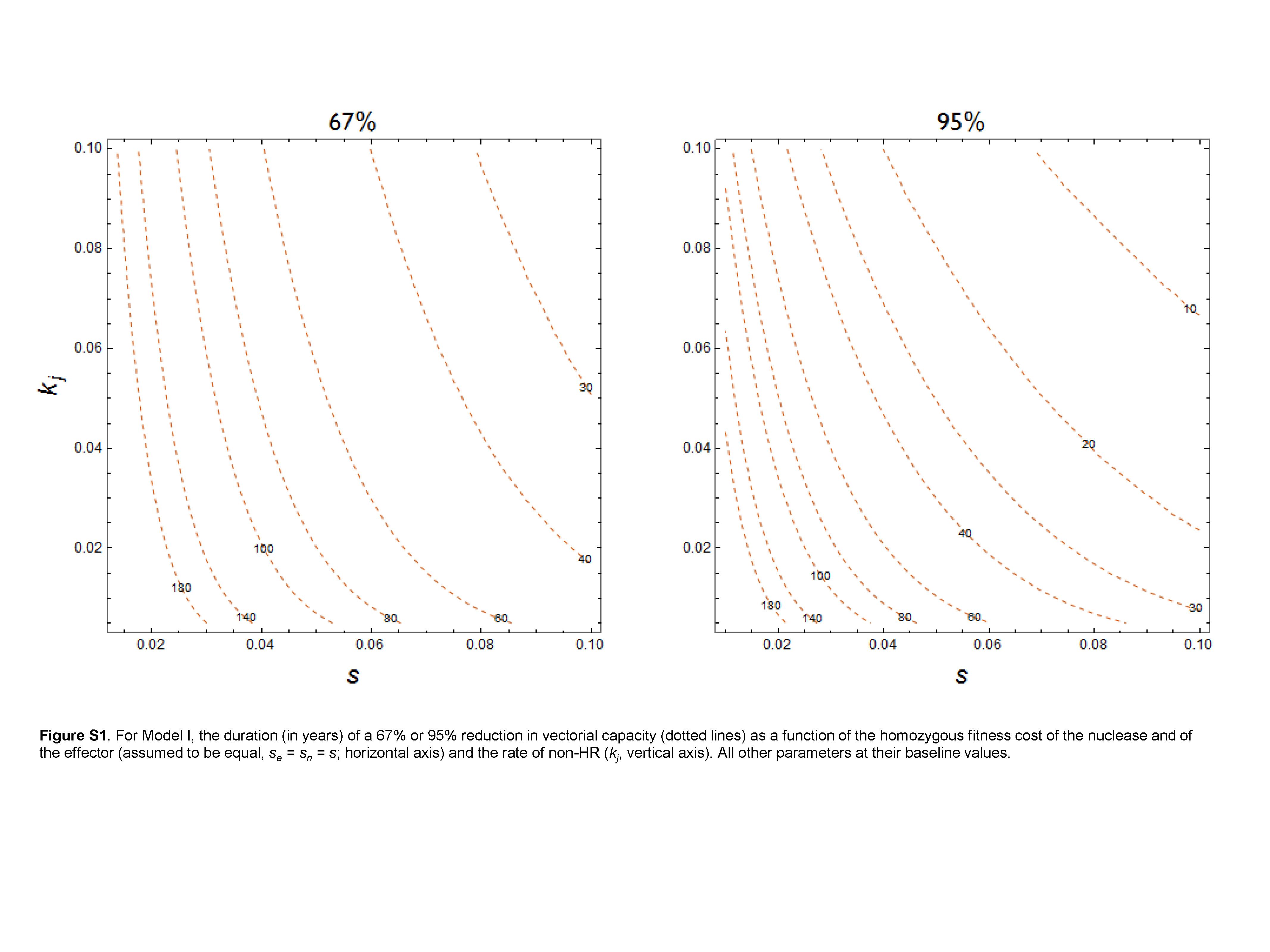

Supplement: Supplementary file 4 [file 1587FigureS1.jpg]

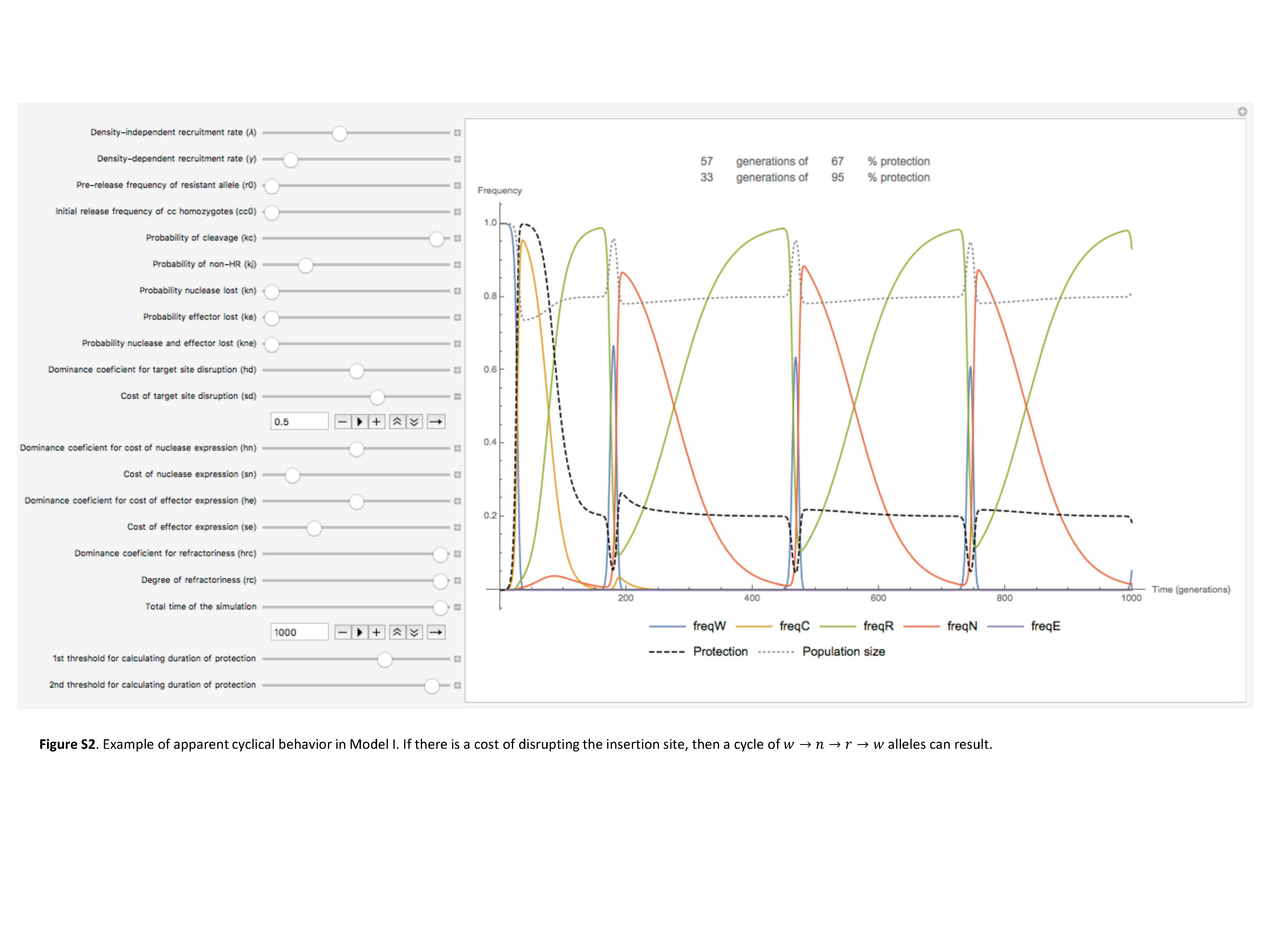

Supplement: Supplementary file 5 [file 1587FigureS2.jpg]
