# Supplementary material for: Requirements for Driving Antipathogen Effector Genes into Populations of Disease Vectors by Homing
Source: Genetics. 2017 Feb 2;205(4):1587–96. doi: 10.1534/genetics.116.197632 (PMC5378115; doi:10.1534/genetics.116.197632)
Supplement: Supplementary file 6 [file 1587TableS1.pdf]

Table S1. The fitnesses of the 15 diploid genotypes and the proportion of each type of gamete produced by them, for Model I.

Diploid genotype	Fitness	Gametes produced				
		w	*c*	*n*	*e*	*r*
ww	1	1	0	0	0	0
wc	$(1- h_d s_d)(1-h_e s_e)(1-h_n s_n)$	$(1-k_c)/2$	$1/2+1/2\ k_c(1-k_j)(1-k_e-k_n-k_{ne})$	$1/2\ k_c k_e(1-k_j)$	$1/2\ k_c(1-k_j)\ k_n$	$1/2\ k_c(k_j+(1-k_j)\ k_{ne})$
wn	$(1-h_d s_d)(1-h_n s_n)$	$(1-k_c)/2$	0	$1/2+1/2\ k_c(1-k_j)(1-k_n)$	0	$1/2\ k_c(k_j+(1-k_j)\ k_n)$
we	$(1-h_d s_d)(1-h_e s_e)$	1/2	0	0	1/2	0
wr	$1-h_d s_d$	1/2	0	0	0	1/2
cc	$(1-s_d)(1-s_e)(1-s_n)$	0	1	0	0	0
cn	$(1-s_d)(1-h_e s_e)(1-s_n)$	0	1/2	1/2	0	0
ce	$(1-s_d)(1-s_e)(1-h_n s_n)$	0	1/2	0	1/2	0
cr	$(1-s_d)(1-h_e s_e)(1-h_n s_n)$	0	1/2	0	0	1/2
nn	$(1-s_d)(1-s_n)$	0	0	1	0	0
ne	$(1-s_d)(1-h_e s_e)(1-h_n s_n)$	0	0	1/2	1/2	0
nr	$(1-s_d)(1-h_n s_n)$	0	0	1/2	0	1/2
ee	$(1-s_d)(1-s_e)$	0	0	0	1	0
er	$(1-s_d)(1-h_e s_e)$	0	0	0	1/2	1/2
rr	$1-s_d$	0	0	0	0	1
