# Supplementary material for: Requirements for Driving Antipathogen Effector Genes into Populations of Disease Vectors by Homing
Source: Genetics. 2017 Feb 2;205(4):1587–96. doi: 10.1534/genetics.116.197632 (PMC5378115; doi:10.1534/genetics.116.197632)
Supplement: Supplementary file 7 [file 1587TableS2.pdf]

Table S2. Transition probabilities due to pre-meiotic germline mutations for each genotype in Model II.

Original genotype	Descendent genotype														
	ww	*wn*	*we*	*wr*	*wd*	*nn*	*ne*	*nr*	*nd*	*ee*	*er*	*ed*	*rr*	*rd*	*dd*
ww	$(1\text{-}m_w)^2$	0	0	0	$2\ (1\text{-}m_w)\ m_w$	0	0	0	0	0	0	0	0	0	m_w^2
wn	0	$(1\text{-}m_n)\ (1\text{-}m_w)$	0	0	$m_n\ (1\text{-}m_w)$	0	0	0	$(1\text{-}m_n)\ m_w$	0	0	0	0	0	$m_n\ m_w$
we	0	0	$(1\text{-}m_e)\ (1\text{-}m_w)$	$m_e\ (1\text{-}m_w)$	0	0	0	0	0	0	0	$(1\text{-}m_e)\ m_w$	0	$m_e\ m_w$	0
wr	0	0	0	$(1\text{-}m_w)^2$	$(1\text{-}m_w)\ m_w$	0	0	0	0	0	0	0	0	$(1\text{-}m_w)\ m_w$	m_w^2
wd	0	0	0	0	$1\text{-}m_w$	0	0	0	0	0	0	0	0	0	m_w
nn	0	0	0	0	0	$(1\text{-}m_n)^2$	0	0	$2\ (1\text{-}m_n)\ m_n$	0	0	0	0	0	m_n^2
ne	0	0	0	0	0	0	$(1\text{-}m_e)(1\text{-}m_n)$	$m_e\ (1\text{-}m_n)$	0	0	0	$(1\text{-}m_e)\ m_n$	0	$m_e\ m_n$	0
nr	0	0	0	0	0	0	0	$(1\text{-}m_n)\ (1\text{-}m_w)$	$(1\text{-}m_n)\ m_w$	0	0	0	0	$m_n\ (1\text{-}m_w)$	$m_n\ m_w$
nd	0	0	0	0	0	0	0	0	$1\text{-}m_n$	0	0	0	0	0	m_n
ee	0	0	0	0	0	0	0	0	0	$(1\text{-}m_e)^2$	$2\ (1\text{-}m_e)\ m_e$	0	m_e^2	0	0
er	0	0	0	0	0	0	0	0	0	0	$(1\text{-}m_e)\ (1\text{-}m_w)$	$(1\text{-}m_e)\ m_w$	$m_e\ (1\text{-}m_w)$	$m_e\ m_w$	0
ed	0	0	0	0	0	0	0	0	0	0	0	$1\text{-}m_e$	0	m_e	0
rr	0	0	0	0	0	0	0	0	0	0	0	0	$(1\text{-}m_w)^2$	$2\ (1\text{-}m_w)\ m_w$	m_w^2
rd	0	0	0	0	0	0	0	0	0	0	0	0	0	$1\text{-}m_w$	m_w
dd	0	0	0	0	0	0	0	0	0	0	0	0	0	0	1
