# Supplementary material for: Requirements for Driving Antipathogen Effector Genes into Populations of Disease Vectors by Homing
Source: Genetics. 2017 Feb 2;205(4):1587–96. doi: 10.1534/genetics.116.197632 (PMC5378115; doi:10.1534/genetics.116.197632)
Supplement: Supplementary file 8 [file 1587TableS3.pdf]

Table S3. Proportion of each type of gamete produced by each descendant genotype in Model II.

Diploid			Gametes	produced	
genotype	*w*	*n*	*e*	*r*	*d*
ww	1	0	0	0	0
wn	$(1\text{-}k_c)/2$	$1/2+1/2\ k_c\ (1\text{-}k_j)\ (1\text{-}k_n)$	0	$(k_c\ k_j\ k_r)\ /\ 2$	$1/2\ k_c\ (1\text{-}k_j)\ k_n+1/2\ k_c\ k_j\ (1\text{-}k_r)$
we	1/2	0	1/2	0	0
wr	1/2	0	0	1/2	0
wd	1/2	0	0	0	1/2
nn	0	1	0	0	0
ne	0	1/2	1/2	0	0
nr	0	1/2	0	1/2	0
nd	0	1/2	0	0	1/2
ee	0	0	1	0	0
er	0	0	1/2	1/2	0
ed	0	0	1/2	0	1/2
rr	0	0	0	1	0
rd	0	0	0	1/2	1/2
dd	0	0	0	0	1
