# Supplementary material for: Requirements for Driving Antipathogen Effector Genes into Populations of Disease Vectors by Homing
Source: Genetics. 2017 Feb 2;205(4):1587–96. doi: 10.1534/genetics.116.197632 (PMC5378115; doi:10.1534/genetics.116.197632)
Supplement: Supplementary file 9 [file 1587TableS4.pdf]

Table S4. Fitness and proportion of each type of gamete produced for each original genotype in Model II (proportions calculated from Tables S2 and S3).

Diploid genotype	Fitness	Gametes produced				
		w	n	e	r	d
ww	1	$1-m_w$	0	0	0	m_w
wn	$(1-h_d s_d)(1-h_n s_n)$	$-(1/2)(1+k_c(-1+m_n))(-1+m_w)$	$1/2(-1+m_n)(-1+k_c(-1+k_j)(-1+k_n)(-1+m_w))$	0	$1/2 k_c k_j k_r(-1+m_n)(-1+m_w)$	$1/2(m_n-k_c(-k_n+k_j(-1+k_n+k_r))(-1+m_n)(-1+m_w)+m_w)$
we	$(1-h_e s_e)$	$(1-m_w)/2$	0	$(1-m_e)/2$	$m_e/2$	$m_w/2$
wr	1	$(1-m_w)/2$	0	0	$(1-m_w)/2$	m_w
wd	$(1-h_d s_d)$	$(1-m_w)/2$	0	0	0	$(1+m_w)/2$
nn	$(1-s_d)(1-s_n)$	0	$1-m_n$	0	0	m_n
ne	$(1-h_d s_d)(1-h_n s_n)(1-h_e s_e)$	0	$(1-m_n)/2$	$(1-m_e)/2$	$m_e/2$	$m_n/2$
nr	$(1-h_d s_d)(1-h_n s_n)$	0	$(1-m_n)/2$	0	$(1-m_w)/2$	$(m_n+m_w)/2$
nd	$(1-s_d)(1-h_n s_n)$	0	$(1-m_n)/2$	0	0	$(1+m_n)/2$
ee	$(1-s_e)$	0	0	$1-m_e$	m_e	0
er	$(1-h_e s_e)$	0	0	$(1-m_e)/2$	$1/2(1+m_e-m_w)$	$m_w/2$
ed	$(1-h_e s_e)(1-h_d s_d)$	0	0	$(1-m_e)/2$	$m_e/2$	1/2
rr	1	0	0	0	$1-m_w$	m_w
rd	$(1-h_d s_d)$	0	0	0	$(1-m_w)/2$	$(1+m_w)/2$
dd	$(1-s_d)$	0	0	0	0	1
